# Experiences of Healthcare Providers Who Provide Emergency Care to Migrant Children Who Arriving in Spain by Small Boats (Patera): A Qualitative Study

**DOI:** 10.3390/children10061079

**Published:** 2023-06-19

**Authors:** María del Mar Jiménez-Lasserrotte, Rosalía Artés-Navarro, José Granero-Molina, Isabel María Fernández-Medina, María Dolores Ruiz-Fernández, María Isabel Ventura-Miranda

**Affiliations:** 1 Department of Nursing Physiotherapy and Medicine, University of Almería, 04120 Almería, Spain; mjl095@ual.es (M.d.M.J.-L.); isabel_medina@ual.es (I.M.F.-M.); md.ruizfernandez@ual.es (M.D.R.-F.); mvm737@ual.es (M.I.V.-M.); 2 Sistema Andaluz de Salud, 18007 Granada, Spain; rosalia.artes.sspa@juntadeandalucia.es; 3 Facultad de Ciencias de la Salud, Universidad Autónoma de Chile, Santiago 7500000, Chile

**Keywords:** child irregular migrant, healthcare providers, emergency care, qualitative research, children’s rights

## Abstract

Background: The phenomenon of migration from regions with more limited resources is a reality of the globalized world. It is estimated that at the end of 2019, almost 80 million migrants were displaced around the world, with 46% of them being children. Almost 20% of the irregular immigrants who arrived in Spain were child irregular migrants, who travelled alone or accompanied by family members after leaving their countries of origin seeking, to find a better future. Child irregular migrants have specific healthcare needs. The objectives of our present study were to describe and understand the experiences of healthcare providers in relation to the healthcare needs and the process of emergency care for child irregular migrants who come to Spain in small vessels. Methods: In this descriptive qualitative study, two focus groups were convened, and in-depth interviews with 21 participants were conducted, followed by inductive data analysis using ATLAS.ti 9.3 software. Results: Three main themes emerged from the analysis: (1) more vulnerable groups for whom the priority is emergency care; (2) the health and social care of child migrants; and (3) challenges and advances in the care of child migrants. Conclusions: For healthcare providers, protecting children, placing value on the family unit, and ensuring that children feel safe at all times are very important. Learning about the experiences of healthcare providers can contribute towards improving the health and social care of children in emergency care.

## 1. Introduction

The phenomenon of migration from regions with more limited resources is a reality of the globalized world [1]. It is estimated that at the end of 2019, almost 80 million migrants were displaced around the world [2], with 46% of them being children. The European Union (EU) has received a third of the world’s migrant population. Many of them are irregular migrants (IMs) who arrived via the ocean to the coasts of Italy, Greece, and Spain [3,4]. The term irregular migrant (IM) refers to “A person who, owing to unauthorized entry, breach of a condition of entry, or the expiry of his or her visa, lacks legal status in a transit or host country” [5]. Most of the IMs who arrive in the EU come from North African and Sub-Saharan African countries [6], from which they embark on a dangerous journey due to economic reasons [7], war, and violence, as well as persecution due to their sexual orientation, politics, or race or for religious reasons [4,8]. Thousands of individuals arrive every year in Spain after crossing the Mediterranean sea in small inflatable boats of 5 to 10 m in length, which are not appropriate for transporting more than 40 or 50 people [9]. The United Nations High Commissioner for Refugees (UNHCR) [10] reported that this dangerous journey has resulted in 15,900 deaths and missing persons in the Mediterranean Sea from 2014 to the present day. Of the 41,861 IMs who arrived in Spain in the year 2020, 96.3% came from Algeria (39.5%), Morocco (20.3%), Mali (12.6%), Guinea (7.6%), the Ivory Coast (6.6%), Senegal (4.8%), or other regions (8.6%). Although most of the IMs who come to Spain are men [9], an increase in the number of women and children has been observed [11]. Almost 20% of the IMs who arrive in Spain are child irregular migrants (ChIMs) [12], who travel of their own volition or accompanied by family members, leaving their countries of origin to seek a better future [13]. ChIMs have specific health needs. They come from countries with severe problems of poverty and hunger, limited access to the healthcare system, and a lack of immunization [14]. During their migratory journey, they stay in camps and forests in Northern Africa in unhealthy conditions with a lack of potable water and food [15,16]. Exposure to diseases, traumas, lesions, or violence [4,9], together with difficulty in accessing health services [17,18], make ChIMs an extremely vulnerable population who need specialized care [19]. Either traveling alone [20,21] or accompanied by their families [22], the safety risks and health problems increase. ChIMs can be invisible to the authorities and can even disappear or become victims of trafficking, work or sexual exploitation, especially girls [20].

ChIMs risk their lives in the ocean as they try to reach Spain, where they are rescued by Maritime Rescue [23,24]. After their arrival on the Spanish coast, they are taken care of by the Red Cross and Security Forces of the state, who provide the response to the emergency [25]. The Red Cross emergency team is composed of doctors, nurses, emergency technicians, social workers, cultural mediators, and human support volunteers, who provide first aid [26]. Healthcare providers perform health and social triage; cover the migrants’ basic needs for hygiene, food, and clothes [25,26]; detect infectious diseases [21] and victims of human trafficking [22,27]; and provide transport to hospitals [25,26]. The health of ChIMs reflects the journey they have taken. They may experience physical exhaustion, dehydration, gastrointestinal problems (vomiting, diarrhea), dermatological problems (chemical burns, scabies), trauma, or respiratory infections [21,26]. Psychological and social stresses also affect their health, with non-specific physical symptoms [28]. During the emergency, healthcare providers try to detect situations of special vulnerability among the ChIMs to offer better support and protection to the children [29]. ChIMs arrive to the new country without documents that verify their age, identity, or relationships with the adults who accompany them [14]. ChIMs need information that is understandable [27], care that is culturally adapted [18,20], psychological care due to the traumatic events of the journey [24], bone tests to determine their age [30], DNA tests to demonstrate their biological relationships [31], and an assessment to detect signs of trafficking [32]. After the emergency care, ChIMs are guarded by the national police, and afterwards, they are taken to host centers or centers for the protection of children [14,20,26]. Some studies have focused on the process of care for ChIMs from the epidemiological [6,12], immunological [33], cultural [18], or legislative [34] perspective or the perspective of the hosting system [35]. Nevertheless, learning about the experiences of the professionals involved in the care process is key for identifying deficits and implementing improvements. In this sense, even though the experiences of ChIMs have been studied through the testimonies of the adults who accompany them [26,36], little is known about the experiences of healthcare providers who provide emergency care to ChIMs as they arrive in boats [9,11,27,37]. The theoretical framework developed by Zimmerman et al. [38] allows us to study the migratory process and the treatment of individuals in different stages and can be applied to study the process of care of ChIMs. The objectives of the present study were to describe and understand the experiences of healthcare providers in relation to the healthcare needs and the process of emergency care for ChIMs who arrive in Spain in small vessels.

## 2. Materials and Methods

### 2.1. Study Design

A descriptive, qualitative design that allows one to describe a phenomenon that is not well-understood, from a realistic perspective, was used in the present study [39]. This approach is adequate for exploring experiences in areas of health from the perspective of healthcare providers who provide care to children who arrive in boats. The Consolidated Criteria for Reporting Qualitative Research (COREQ) were followed [40].

### 2.2. Participants and Setting

The study was conducted at the Spanish Red Cross offices. The participants were selected though intentional sampling if the following criteria were met: a health or social professional in the emergency team, minimum experience of one year in emergency care, and provision of informed consent. The exclusion criterion was decline to participate in the study. To recruit the sample, one of the researchers had a prior meeting with those in charge of the institution, after which the healthcare providers were invited to participate through a telephone call, and a meeting was set up. In total, 24 healthcare providers were invited to participate, of which 3 declined to participate due to a lack of time. The final sample was composed of 21 healthcare providers. The sociodemographic characteristics of the participants are shown in Table 1.

### 2.3. Data Collection

The collection of data included 21 participants. Two focus groups (FGs) were assembled, each composed of five healthcare providers, and eleven in-depth interviews (IIs) took place between October 2022 and January 2023. Both the FG meetings and IIs took place in the meeting rooms of the Spanish Red Cross. The FG meetings lasted for an average of 62 min. Each participant gave an individual interview, with a mean duration of 43 min. The FG meetings and IIs were conducted by three researchers, following a semi-structured interview guide (Table 2). Before starting the session, the researchers collected the sociodemographic data, explained the protocol, and guaranteed the confidentiality of the data, and the participants signed the consent form. The interviews were conducted in Spanish, recorded in audio, and transcribed verbatim. At the end, the participants had the opportunity to read the transcripts to verify the content. The collection of data ended when data saturation was reached.

### 2.4. Data Analysis

The IIs and FC meetings were independently transcribed and analyzed with the computer quantitative analysis program Atlas.ti 9.3 by two researchers, together with the annotations from the researchers. The thematic analysis was performed following the phases described by Braun and Clarke [41]: (1) Become familiar with the data: transcription, reading and re-reading of data, and make notes of the initial ideas; (2) generation of initial codes: systematic coding of the data groups; (3) theme search: conversion of the codes into themes; (4) review of themes: verify the fit between codes and themes; (5) definition/designation of themes: analyze and refine the details of each theme; and (6) create a report: select examples of themes and sub-themes, align the analysis with the research question, and create a final report. 

### 2.5. Rigor

To ensure rigor, the criteria proposed by Guba and Lincoln were adapted [42]. Credibility: The participants were familiarized with the setting to establish trust and obtain valuable data. A triangulation of researchers was used for decisions on coding, analysis, and interpretation. Transferability: A detailed description of the experiences and context of the study was established. Reliability and confirmability: The transcriptions were created by nurse researchers, revised by members of the research team, and verified by the participants, who approved them and did not propose changes. The transcriptions were utilized as an audit trail to document the decisions. Reflexivity: A small diary allowed the researchers to examine their own conceptual lenses, values, and preconceptions.

### 2.6. Ethical Considerations 

The study was conducted following the ethical standards of the Declaration of Helsinki. Approval was obtained from the Spanish Red Cross Ethics and Research Committee (grant number: CR-20-01). Before starting the study, a written consent form and permission to record the interview were obtained.

## 3. Results

Inductive data analysis was used to extract three main themes and eight subthemes, which helped us to describe and understand the experiences of the healthcare providers regarding the healthcare needs and the process of emergency care of the ChIMs who come to Spain in small vessels (Table 3).

### 3.1. Most Vulnerable Groups: Priority of Care during the Emergency

ChIMs are an especially vulnerable collective. Healthcare providers start to act as soon as the Maritime Rescue teams transport the rescued individuals to the port. From the start, a prioritization process of the individuals is performed, starting with the most vulnerable groups, such as children, the injured, and pregnant women. Next, a sociosanitary triage is performed, and the care needed is provided. The emergency team is multidisciplinary, composed of different professionals so as to tend to all the needs of the ChIMs, with coordination among the different institutions involved in the process of care being indispensable. 

#### 3.1.1. Ready for an Emergency: Action Protocols

Before emergency care, the healthcare professionals coordinate with Maritime Rescue, in charge of rescuing individuals in danger in the ocean, who provide information about the number of individuals rescued and the health conditions with which they arrive. When the rescue vessel arrives at the port, the healthcare providers are ready at the pier with all the materials and human resources necessary to provide support during the disembarkment of the newly arriving migrants.

“The first to disembark are the children, pregnant women, and the injured. The team leader sizes the needs, because we know that when we have nursing babies, once they give them to you, you cannot help in the disembarkment until the mother disembarks”. (FG1-2)

Afterwards, they are accompanied to the care modules, which are divided into different areas: triage, a shower area, and a nurse station. All the new arrivals progress through triage with a nurse and a cultural mediator, which enables the detection of initial health problems and a social assessment to evaluate the reasons for the migrants’ arrival and their family situation and to detect vulnerabilities. As one of the participants stressed:

“A health triage is performed to learn about their state of health, if they vomited, if they had diarrhea, their temperature is taken…” (FG2-1)

“The triage we perform is sociosanitary triage, and it is just as important… a lot of importance is granted to it”. (I-3)

The healthcare providers provide comprehensive care that includes all the health, social, and emotional aspects that can create discomfort for the individuals. As a limited intervention time is available, care is prioritized to these collectives, assessing the children continuously.

“For example, a child who is withdrawn, more afraid, they are monitored, he or she is recorded…it could be that he or she is tired, or he or she may have a problem that was not shown during the initial triage”. (FG1-2)

#### 3.1.2. Covering Basic Needs

After triage, the children’s basic health needs are covered. The ChIMs go to the shower area, and depending on their age, they go alone or accompanied by a family member. They are provided with clothes according to their age, as well as personal goods they may need. As one participant stated, the ChIMs are accompanied throughout the entire process:

“The first we try is for them to access a hot shower, especially when they arrive injured or with wounds, many times we have to help moms with their baby’s hygiene because they are exhausted”. (I-7)

If they do not need healthcare, after showering, they are taken to the children’s room, where they are offered restorative food, considering their age, allergies, or other food intolerances they may have.

“I remember that one time we had a celiac child, and we did not have any problems. In the warehouse, we had food without gluten, lactose, or other allergies”. (I-8)

The children’s room has games and educational resources, which allow the health providers to communicate and establish links that allow the ChIMs to forget the situation experienced. The individuals in charge of this function receive specific training on caring for children in emergency situations. As various participant explained, it is important to cover the emotional and leisure needs of the children, always maintaining their family structures.

“For us, the protection of children is very important, as well as the family unit, and that they feel safe. In the children room, you can observe family relations, emotional reactions…because they are part of a much unprotected group”. (I-7)

#### 3.1.3. The Multidisciplinary Healthcare Team and Coordination with Other Institutions

The emergency team is a large multidisciplinary team. It must be highlighted that within the team, the cultural mediators know English, French, and Arabic perfectly, even including some different dialects. The closeness and sociocultural aspects are also important, as they allow for the detection of cultural elements necessary for good care.

“There are small gestures, small habits that could go unnoticed, if a person has an amulet tied to the waist, we must know that it is a culturally valuable amulet, or a religious one, and we try to respect all the cultural beliefs”. (I-5)

The mediators counsel the healthcare providers so that they are able to provide culturally adapted care. Additionally, they are also specialists in the detection of vulnerabilities, with basic knowledge on Spanish rights and legislation about international protection. Based on the vulnerability signs detected, an in-depth interview is performed to collect the information needed to start the necessary paperwork and to coordinate the rest of the resources for the monitoring of the most vulnerable cases, such as possible victims of human trafficking.

“Both the reasons for their migration and the clandestine mode of travel leads to helplessness, institutional lack of protection and violation of rights. It is important to collect all of this information to communicate it to the institutions responsible, they are more susceptible to being recruited by human traffickers”. (I-10)

The national police are present during the emergency care. The ChIMs arrive in an irregular manner and are escorted and received for police report. Coordination with other institutions is important, and the cases of the ChIMs are transferred to the Brigade of Alien Affairs, Juvenile Prosecution Services, or the hosting services where they can be sent.

“When we finish the intervention, the administrative procedures begin. The team leader transfers the health information and other vulnerabilities to the police, social reports are sent to the administrations and entities in charge of providing healthcare resources and protection to the children”. (I-11)

### 3.2. Health and Social Care of Migrant Children

After health and social triage, the ChIMs who need health care are transferred to nursing services so that the nurses can provide care. Next, a social assessment is performed to detect vulnerabilities. The children’s rights are taken into consideration, as well as the existence of family links, or lack thereof, with the person who accompanies them and other pertinent paperwork needed after their arrival in the host country.

#### 3.2.1. Main Health Problems

The health assessment performed includes a complete clinical examination based on the triangle of pediatric evaluation. An assessment of the state of circulation, respiration, and the appearance of the child is performed. The health providers take into account characteristics according to age, level of development, and country of origin. Many of the ChIMs come from developing countries with limited access to healthcare systems, insufficient vaccination coverage, and malnutrition.

“If they are nursing, we look at their belly button, if they are newborns, we look at the fontanelles, their hydration status, which in children is very important”. (FG2-1)

During their migration journey, these children are exposed to adverse climatic conditions, dehydration, and malnutrition due to the lack of food and water. They tend to have dermatological problems due to fungi and parasites as a consequence of their stays in unhygienic places. They may also have traumas from the conditions of the journey, such as cuts or lesions on their feet due to walking without shoes.

“Interestingly, the pathologies we observe are not extremely severe (…), the children are highly resilient. ¿They tend to come with a cold, diarrhea, vomiting, and many insect bites, as they stayed in the forest before coming”. (FG2-3)

The journey across the ocean in a “patera” (open boat) can have a detrimental effect on the children’s health. The health consequences of a journey in a patera will depend on the duration, the meteorological conditions, and the overcrowding experienced during the journey. The main health problems detected due to the journey in the patera included general malaise, headaches, burns, colds, and dehydration. The study participants highlighted that despite the harshness of the journey, they generally arrived in a good state of health.

“I have taken care of chemical burns, especially in the areas of the buttocks and legs. They are produced by the mixing of seawater and gasoline, which impregnates their clothes, resulting in these lesions”. (I-1)

“It is very important to hydrate them. They arrive after many hours without drinking much water and under a prolonged exposure to the sun. The first thing we do is to monitor the state of hydration of the children”. (FG2-4)

The migration journey exposed the ChIMs to adverse situations that led to stress and anxiety, which affected their psychological state. During the journey, ChIMs may experience extreme situations of hunger, cold, sexual abuse, and violence, and they may witness the deaths of family members and friends. It is probable that these situations of post-traumatic stress will lead to nightmares, fears, or somatic manifestations.

“I remember a 5 year-old child who did not want to shower, he cried a lot. His mother told us that he was afraid of water due to the journey in the boat”. (I-7)

“During the journey on the boat, they experience very critical moments, and sometimes the children are given drugs to make them sleep, so that they do not move much”. (FG2-2)

#### 3.2.2. Social Care of Children Who Are Not Accompanied

ChIMs are an especially vulnerable group. Their situation can become more aggravated if they travel alone, without an adult who can protect them. It is important to identify the specific needs of the children, provide information about their rights, offer new resources of support as necessary, and coordinate the entities involved. For healthcare providers, this is a very complex task, given the joint arrival of very different migrants and their lack of documentation, which impedes the verification of their identities or their real ages. Age is a decisive factor, as it will determine whether the child qualifies for state guardianship.

“It is not strange to find children who travel alone, they tend to have an age threshold of 15–17 years old. However, sometimes we have helped younger children, from 8 to 10 years old who travelled alone”. (FG1-3)

“They have to wait until the test results, and then if the decision is made that he or she is a minor, then he or she will be transferred to child protection services”. (FG1-4)

If the child’s underage status is questionable, the prosecutor is contacted, who decides if a test needs to be performed to determine their age. This test consists of an X-ray of the left hand that shows bone maturation, which is considered as a reflection of biological age. However, these tests are not very precise, and on occasion, some ChIMs have been declared adults and, therefore, not been able to access child protection services. These ChIMs are transferred to a host center or an Alien Internment Center.

“The bone test has a large margin of error, and racial, nutritional, psychological, or cultural aspects are not taken into account, which could have an influence on the development and growth of children”. (I-4)

ChIMs who are not accompanied tend to have their own migrant journeys. They tend to have a complex socio-economic situation and no family support. Some escape from their countries due to harmful practices such as the genital mutilation of females or forced matrimony. During the migrant journey, they may experience different adverse situations from living under clandestine conditions, border crossings, or street living situations. With respect to the question of gender, many girls declare that they are adults after their arrival in the new country, and age tests are not usually performed, so that they are left unprotected and within reach of trafficking networks.

“We are especially worried about girls who travel alone, because many of them have suffered sexual abuses and violence during the journey…You try to explain to them about the danger of being out of reach of child protection networks, but it is difficult to fight against the trafficking networks”. (I-11)

#### 3.2.3. Social Care of Children Accompanied by Adults

Most of the family units travel without documents that verify their family relationships. Many of these families travel to improve their living conditions or escape harmful practices that are practiced in their countries. The healthcare providers try to learn about the family’s documentation situation and detect any vulnerability to provide this information to the juvenile prosecution services. During the healthcare process, the health professionals try not to separate the families and try to observe signs of family bonds.

“I remember a dad who arrive on the patera with his son, he did not leave him alone at any moment. The child had cancer, he had the health documentation so that the child could be taken care of at a hospital near here”. (I-6)

Some families are provided information about the DNA test to demonstrate the biological link, a report is created about the family unit, and they are sent to a host center while they wait for the results. On some occasions, the children are separated from their mothers by the mafia and are returned so that they can enter the host country together. In cases of suspicion, unjustified rejection of the test may be a basis for the solicitation of judicial measures for the protection of the child, who will then be under the guardianship of the state.

“It is a very complex situation, most arrive without documents, and without a family book, with children who were born during the migratory journey, and who do not have a birth certificate. On some occasions, the mafias separate the mothers from the children, but you do not see a bond between them…it’s very complicated because you can blame a victim”! (I-3)

On some occasions, the adult who accompanies the child does not have a blood tie with the child but a sentimental bond, although this cannot be verified formally. This situation is more complicated, because the adult and child can be negatively impacted by an added vulnerability. On most occasions, there are ties that bind them, with this being a positive factor for the child during the journal and disembarkment. There is discrimination toward the male gender, especially if they are accompanied by a male adult. Cases of separation of the child until proof of a paternal association is provided with genetic results have been observed.

“Many children come accompanied by the extended family, an aunt, a friend of the family, who took care of the child because the mother died, because the parents asked them to bring them to Spain, but he or she cannot prove this. The separation is very bitter for the child”. (FG1-4)

“I remember a father who came with his daughter. They were separated as soon as they arrived. The girl was sent to a center for children, and the father to an Alien Internment Center. When the positive blood ties results came back, the father had already been deported to his country of origin”. (I-4)

### 3.3. Challenges and Advances in the Care of ChIMs

Most of the people who form part of the team are volunteers, who dedicate their free time to perform altruistic tasks. The participants feel lucky for being able to perform this voluntary work, which has an effect on their personal life, they feel that the ones who benefit are them, not the migrants. However, their values go against the values of others who are less aware about this collective.

#### 3.3.1. Awareness versus Social Prejudices

Healthcare providers are volunteers who balance this work with their other work outside the institution. The study participants highlighted this labor as enriching, because it provided them with growth in their personal and working lives, changing their perceptions of life.

“For me, it has led to important personal growth. I do not’ have any obligations to go, I go because I want to, for my colleagues, and because I like being part of a team”. (FG1-2)

Working with migrants allowed them to break away from pre-established stereotypes and to understand the reasons and needs associated with embarking on this journey. On the personal level, one of the participants highlighted that it conferred on them a much broader view of the world, a great awareness, and the ability to empathize with others.

“Sometimes we forget that we are also immigrants when we leave, that we have also felt the need to improve our lives (…) a right of wanting a better life for one’s own, is an inherent human right”. (FG1-3)

This migration phenomenon has resulted in a feeling of rejection among some sectors of society. The situation of irregularity, giving a person the epithet of “irregular”, leads to a negative view of this collective that is disseminated through communication media. On many occasions, this information is not verified and xenophobic opinions are manifested that are erroneous or hoaxes and are disseminated among the population, resulting in greater rejection of this collective and creating ethical conflicts among the participants.

“Ultimately, there is more rejection. There are people who have pre-conceived ideas and some stigmas that perhaps have nothing to do with what is coming or what is truly happening”. (FG2-5).

#### 3.3.2. Child Protection: Advances in Social and Health Care

The arrival of ChIMs whose underage status is suspicious was presented by the study participants as a challenge that must be improved. A protocol to determine age that includes a psychological assessment, for which the children can remain in the center while they wait for the results, could be implemented. During this appeal, they could talk to a lawyer and feel welcomed and understood.

“I believe that this would improve the legal advice provided, which does not depend on us, but on the lawyer on duty”. (I-3)

As for ChIMs who are accompanied, a need to improve protocols was detected. Many of these ChIMs are separated from their families as they wait for the genetic results, which can take a few months. On other occasions, they are sent to the hosting centers with the adults who accompanied them, and they leave the center before the test results arrive. As one of the participants highlighted:

“They can leave the center whenever they want, they reject their spot. Sometimes we get negative DNA results, but we do not know where the children are, we lost them”! (I-11)

With respect to health matters, the participants underlined the inherent problems in the case of a hospital transfer. For healthcare providers, this is a severe problem, as they have to go back to the emergency situation, and there is no one available in the hospital who could accompany the child. Additionally, a language barrier will be added to this problem.

“Where there is child with a health problem we take him or her to the hospital. Then, we have problems to find a translator, and finding someone to be responsible for the child”. (I-2)

The ChIMs remain under police custody for a maximum of 72 h. In cases where a medical recommendation is prescribed to the ChIMs, it is difficult to continue with their care. The participants indicated that they were left under the care of the police, who, among many other functions, had to provide medication or follow the guidelines according to the changes in the diseases. As one of the participants stated, they did not have a family doctor or health card.

“When a child has to go to the hospital because of chest wheezing, and they are told: “follow up with your family doctor in 48 h”, we know that it’s impossible”! (FG2-4)

## 4. Discussion

The objective of the present study was to describe and understand the experiences of healthcare providers related to the health needs and the process of emergency care of ChIMs who arrive in Spain in small vessels. The theoretical framework of Zimmerman et al. [38] allowed us to study the conditions experienced by the ChIMs during the different stages of the migratory journey.

### 4.1. Most Vulnerable Groups: Priority of Emergency Care

Thousands of IMs arrive in Spain through the Western Mediterranean route in small vessels from Northeastern Africa [11,26]. The population that travels through this dangerous route is mainly composed of men, with children being the second most numerous group [14,43]. The ChIMs abandon their countries of origin in search of a better life or escaping from rights violations and poverty [20]. In cases where ChIMs travel alone, the risks and vulnerabilities increase [4]. During their transit, the ChIMs live in forests and camps in unhealthy conditions, with limited access to basic food, potable water, and the health system [4,16]. They can also experience anxiety, stress, and other psychological problems due to their witnessing of, or exposure to, situations of violence [21,24]; many become victims of sexual exploitation or forced labor [20]. ChIMs embark on dangerous journeys in “pateras” in overcrowded conditions, in fear or sedated [26]. The maritime rescue teams prioritize the rescue of ChIMs, need special care, and are transferred to the closest port to be taken care of by emergency teams [24]. After their arrival in Spain, their right to healthcare is guaranteed through the organization of volunteers such as the Red Cross [4,14,25]. These teams are composed of healthcare providers, who provide first aid, as well as health and social triage that is culturally adapted, and provide coverage of their basic needs [18,26]. In agreement with other studies, children have the right for their basic needs to be satisfied [20]. The care of children is based on the provision of high-energy foods, hygiene, and leisure to provide safety and socio-emotional well-being [26,44].

### 4.2. Health and Social Care of Migrant Children

The health of newly arrived ChIMs is reflects the journey they have taken, which includes risks to their physical and mental well-being [21,43]. Our results agree with those of other studies, in that the ChIMs need health care due to dehydration, malnutrition, dermatological and gastrointestinal problems, and chronic diseases [24,26,43]. They have high rates of post-traumatic stress and psychological anguish due to the traumatic events experienced or their separation from their families [21,43]. During the caregiving process, the healthcare providers try to not to separate the ChIMs from their parents, observing the family ties and avoiding negative impacts on the children [14,26]. Other matters addressed in the study results were the detection of social vulnerabilities and children’s rights. The tests used to determine the age of the ChIMs indicate that tending to physical factors alone is not enough to assess age, and other psychological, social, or cultural factors are also needed [30,44]. Coinciding with the Association for Human Rights of Andalusia (APDHA) [29], on some occasions, the ChIMs say that they are adults to able to continue with their migrant journey. In the case of adolescent girls, they become more exposed to recruitment by human trafficking networks, and they are excluded from the protection system [45,46]. Many family units travel with documents that can verify their family ties. In agreement with other studies, the future of these families will depend on the decisions of the authorities as a function of the genetic proof from the DNA tests that are performed within the context of migrations [14,47]. These tests only analyze genetic markers that assess close relationships such as father–son bonds, excluding the extended family [37]. The protection of ChIMs in centers of protection will be implemented when the tests are rejected or there are signs of risks for the child [14]. 

### 4.3. Challenges and Advances in the Care of Migrant Children

However, the application of these guidelines requires improvements that consider that family relationships do not always correspond to genetic ties in every culture, an increase in the speed of the test results’ processing, or actions in the case of father–daughter relations [37,48]. The results of the study highlight the importance of having cultural mediators and child specialists with training in rights and legislation so that the ChIMs receive adequate information to overcome cultural barriers and lack of trust and for their transfer to the child protection service [20,31,35,44]. According to the UN Convention, ChIMs have the same right to health services as any other child. However, the results show that they face care with a lack of interpreters, transcultural care, and interruptions in the continuity of their care [4,43,48]. The legal status of ChIMs should be separated from emergency care, resolving problems of coordination with police to provide adequate health care [49,50]. The condition of “irregular migrant” reinforces the xenophobic behaviors and rejection exhibited by some sectors of society, which have impacts on the health and integration of ChIMs [51]. Providing emergency care with a holistic approach to ChIMs requires commitment and cultural training [50,51,52]. Social prejudices become ethical conflicts for the healthcare providers who perform fundamental work in the care of ChIMs [1,26]. The evidence from our study reveals that learning about the experience of the healthcare providers involved in the care of the ChIMs can contribute towards the improvement of action protocols, as it provides more specialized answers.

### 4.4. Limitations

All the participants perform their voluntary work in the same institution in southern Spain. Recruiting participants from other institutions or countries could change the results.

## 5. Conclusions

Healthcare providers begin to act as soon as the Maritime Rescue teams transport the people they rescue to the port. The resources, although limited, are broad, allowing for comprehensive and efficient care according to the needs detected. Once the detection of the main problems with which the migrants arrive is complete, the healthcare providers provide comprehensive care that includes all the health, social, and emotional aspects that could create discomfort for the children. For healthcare providers, the child’s protection is very important, and value is placed on the family unit, as well as the children’s feeling of safety. The work performed by the mediation team is fundamental for communication between all the healthcare providers and the migrants.

Healthcare providers work in a coordinated manner with different institutions. Coordination with police and children protection services is important for transferring all the relevant information. It is important to detect the specific needs of the children to provide them with information about their rights, offer support resources as necessary, and coordinate with all the entities involved. For healthcare providers, this is a very complex task, as in the same vessel, the healthcare providers may find many different types of migrants. Girls have greater vulnerability due to their increased risk of experiencing sexual violence, and this matter of gender is of special concern for healthcare providers.

Volunteering is an activity that healthcare providers combine with their work outside of the institution. This work provides them with opportunities for growth in their personal and working lives, changing their perceptions of life.

## Figures and Tables

**Table 1:** Sociodemographic characteristics of the participants (*N* = 21).

Participant	Age	Nationality	Marital Status	Sex	Role in Emergency Team	Years of Experience
FG1-1	38	Senegal	Married	Male	Cultural Mediator	12
FG1-2	27	Spain	Single	Male	Early Childhood Specialist	10
FG1-3	57	Spain	Single	Male	Team Leader	12
FG1-4	35	Spain	Single	Female	Social Worker	8
FG1-5	38	Morocco	Single	Female	Cultural Mediator	12
FG2-1	24	Morocco	Single	Female	Nurse	5
FG2-2	40	Spain	Single	Female	Nurse	12
FG2-3	36	Spain	Married	Female	Nurse	10
FG2-4	38	Spain	Married	Male	Nurse	12
FG2-5	35	Spain	Single	Female	Nurse	10
I-1	39	Spain	Single	Female	Nurse	8
I-2	60	Spain	Married	Female	Nurse	6
I-3	50	Spain	Divorced	Female	Cultural Mediator	12
I-4	39	Spain	Married	Female	Social Worker	8
I-5	58	Spain	Married	Female	Cultural Mediator	7
I-6	37	Spain	Married	Female	Nurse	9
I-7	35	Morocco	Single	Male	Early Childhood Specialist	6
I-8	35	Spain	Single	Male	Team Leader	6
I-9	30	Spain	Single	Male	Early Childhood Specialist	4
I-10	48	Morocco	Married	Male	Cultural Mediator	15
I-11	39	Spain	Single	Female	Social Worker	13

FG: Focus group; I: In-depth interview.

**Table 2:** Interview protocol.

Stage	Subject	Content/Possible Questions
Introduction	My intention	Learn about the experiences of the healthcare professionals who provide care to migrant children who arrive in small vessels.
	Ethical issues	Provide information about voluntary participation, recording, consent, confidentiality of the data, and the option of leaving the study at any time.
Beginning	Introductory question	Could you talk to me about your experience in providing care to migrants who arrive in small vessels?
Development	Conversation guide	Could you discuss how you organize yourselves to deal with emergency situations? What are the main needs with which the children arrive? Tell me how you coordinate with other institutions. Could you explain to me what the main limitations in the care provided are? What is your opinion on the perception of migration in the society in which you live?
Closing	Final question	Is there anything else you would like to tell me?
	Appreciation	Thank them for their participation, remind them that their interview will be of great use, and place yourself at their disposal.

**Table 3:** Themes, sub-themes and units of meaning.

Theme	Sub-Theme	Units of Meaning
Theme 1. Most vulnerable groups: priority of emergency care.	Ready for an emergency: action protocols	The first to be rescued, social and health triage, team actions, continuous assessment.
	Covering basic needs	Children space, shower, hygiene and food, pediatric-specific materials, leisure needs, games, children area.
	The multidisciplinary healthcare team and coordination with other institutions	Resources, social mediation, multidisciplinary team, national police, juvenile prosecution service.
Theme 2. Health and social care of migrant children	Main health problems	Wounds, burns, dehydration, vomiting, fear, tiredness, phobias.
	Social care of children who are not accompanied	Loneliness, age tests, doubtful underage status, gender perspective, trafficking.
Theme 3. Challenges and advances in the care of migrant children	Social care of children accompanied by adults	No documents, DNA tests, family ties, discrimination according to sex.
	Awareness versus social prejudices	Awareness, empathy, negative perspective, rejection
	Child protection: advances in social and health care	Social and judicial counseling, complementary tests, continuity of care, hospital transfers, medicine.

## Data Availability

The data presented in this study are available on request from the corresponding author.
